# Radiation oncology education and experience in the undergraduate medical setting

**DOI:** 10.1080/10872981.2021.1899643

**Published:** 2021-03-15

**Authors:** Otasowie M. Odiase, Danning Huang, Karna T. Sura

**Affiliations:** a Department of Radiation Oncology, Upstate Medical University, Syracuse, NY, USA; b Department of Public Health and Preventive Medicine, Upstate Medical University, Syracuse, NY, USA

**Keywords:** Radiation oncology, education, undergraduate medical education

## Abstract

The goal of this study was to determine the impact and experience of radiation oncology (RO) education in the undergraduate medical experience in the USA. A list of American medical schools was complied from various sources including the Association of American Medical Colleges (AAMC) and American Association of Colleges of Osteopathic Medicine (AACOM) in the summer of 2019. Data was extracted through institution website review, individual phone calls and email distribution. A total of 198 programs (155 allopathic and 43 osteopathic medical schools) were included. Every medical school curriculum had oncology lecture during MS year 1 and 2, although a minimal amount (4%) had a RO-specific lecture during MS year 1 and 2. There were significant differences in the RO education and experience in allopathic (MD) versus osteopathic (DO) programs. Home radiation oncology programs and career advising were associated with a radiation oncology elective during year 3 and 4. Furthermore, RO career advisors and older schools were associated with having one student match into radiation oncology. RO education during the didactic portion of the undergraduate medical experience remains extremely limited. This limitation is even more pronounced in medical schools without RO mentorship and in osteopathic medical schools. This lack of RO exposure perpetuates itself by bringing less students into the field. These issues require attention both on a national and medical-school-specific level.

## Introduction

Cancer is the second leading cause of death worldwide, and has an impact on almost every field of medicine [1].

While several medical specialties may be involved in the care of cancer patients, oncologists, radiation oncologists, and surgeons remain the three main pillars of cancer treatment in medicine. Radiation treatment is used for the treatment of approximately 50% of all patients with cancer and accounts for over 40% of the cure rates [2].

Previous studies have demonstrated a lack of oncology education in the undergraduate medical setting [3–5]. Furthermore, radiation oncology (RO) remains a small proportion of the oncology educational experience for medical students. Various programs have attempted to increase exposure to RO are in stages of development and/or implementation, yet it is unknown how effective these have been [3,6].

The goal of this study was to determine the impactof RO education and experience in the undergraduate medical experience in the USA.

## Method

A list of American medical schools was complied from various sources including the Association of American Medical Colleges (AAMC) and American Association of Colleges of Osteopathic Medicine (AACOM) in the summer of 2019. After the initial list was created, schools with multiple campuses were consolidated into one location. Medical schools that were recently established and had no graduating class were excluded from the analysis. A total of 198 programs (155 allopathic and 43 osteopathic medical schools) were included in this analysis.

Data was extracted from various sources including the school’s website, telephone calls, and emails. Key characteristics were collected including MD or DO program, RO program with or without residents, RO specific interest group, oncology lectures including RO-specific lecture during MS1-2, RO rotaitons electives during MS3-4, career advisors for RO, and match into a RO residency program.

Frequency and proportion were used to summarize the distribution of key characteristics. Data was analyzed to evaluate the association of (1) having a 3rd or 4th year elective in radiation oncology and (2) matching in radiation oncology. Pearson chi-square test was used, or Fisher exact test was used if more than 25% of cells have expected counts less than 5. Multivariate logistic regression was further applied to confirm the association while adjusting the confounding effects from other related factors. The data analysis for this paper was generated using SAS software, Version 9.4 (SAS System, Cary, NC, USA). *P*-value of <0.05 was considered statistically significant.

## Results

A total of 198 medical schools were evaluated. Table 1 lists the general summary of the data obtained. A majority of medical schools had a home RO department (58.1%), but a minority had a department with RO residents (42.4%). Every medical school curriculum had oncology lecture during MS year 1 and 2 although a minimal amount (4%) had a RO-specific lecture during MS year 1 and 2.

**Table 1.** General summary

Category	Subcategory	*n* (%)
MD or DO		
	MD	155 (78.3%)
	DO	43 (21.7%)
Has a home radiation oncology program?		
	Yes	115 (58.1%)
	No	83 (41.9%)
Has a home radiation oncology with radiation oncology residents?		
	Yes	86 (42.4%)
	No	112 (56.6%)
Has a general oncology interest group?		
	Yes	89 (45.0%)
	No	103 (52.0%)
	Unknown	6 (3.0%)
Has a RO specific interest group?		
	Yes	21 (10.6%)
	No	171 (86.4%)
	Unknown	6 (3.0%)
Has Oncology lecture during MS1-2?		
	Yes	198 (100%)
	No	0 (0%)
Has a RO oncology lecture during MS1-2?		
	Yes	8 (4.0%)
	No	189 (95.5%)
	Unknown	1 (0.5%)
Has a RO elective during MS3-4?		
	Yes	121 (61.1%)
	No	77 (38.9%)
Has career advisor for RO?		
	Yes	129 (65.2%)
	No	69 (34.8%)
Has a match in radiation oncology residency?		
	Yes	154 (77.8%)
	No	31 (20.7%)
	Unknown	3 (1.5%)

Abbreviations: RO (Radiation oncology), MS (Medical school).

There were significant differences in the RO education and experience in allopathic (MD) versus osteopathic (DO) programs. Overall, RO didactic lectures during the first two years of medical school were minimal all around (~4% of all medical schools). DO programs were less likely to offer both a RO elective (*p* < 0.001) and have a career advising for RO (*p* < 0.001) than MD programs. With regards to residency match, 94% of MD programs match at least one graduating medical student into RT compared with just 25% of DO programs (*p* < 0.001). There was no significant difference in RO lectures given during the first year (5.2% in MD programs versus 0% in DO programs, *p* = 0.204).

We examined whether there was an association between having a RO lecture during the pre-clinical years of medical school and the number of students who chose to pursue a RO elective during their clinical years. There was no significant association, but programs with a home RO program with residents did trend significantly (*p* = 0.078). Table 2 demonstrates the significant associations with medical schools that have a radiation oncology elective during year 3 and 4. Multi-variate model revealed having a home radiation oncology program had an odds ratio of 12.62 (4.60–34.60), as well as a career advisor for radiation oncology had an odds ratio of 2.95 (1.34–6.50) of having a radiation oncology elective during year 3 and 4.

**Table 2.** Univariate analysis of having a 3rd or 4th year elective in radiation oncology

Category		No	Yes	*P* value
Year established				<.001
	Before 1981	42 (54.55)	100 (82.64)	
	Since or after 1981	35 (45.45)	21 (17.36)	
Has home rad onc program?				<.001
	No	58 (75.32)	25 (20.66)	
	Yes	19 (24.68)	96 (79.34)	
Has career advisor for RO?				<.001
	No	50 (64.94)	19 (15.70)	
	Yes	27 (35.06)	102 (84.30)	
Has Home Radiation Oncology program with residents?				<.001
	No	71 (92.21)	41 (33.88)	
	Yes	6 (7.79)	80 (66.12)	

A majority of medical schools (77.8%) had at least one graduating medical student match into RO residency. Univariate analysis of this data is in Table 3. Multi-variate analysis demonstrated that medical schools established before 1981 had an odds ratio of 12.14 (4.26–34.55) of having a student match into RO. Also, having a RO career advisor available during medical school had an odds ratio of 31.84 (8.58–118.12) of having at least one medical student match into RO.

**Table 3.** Univariate analysis of having someone match into radiation oncology. *P* < 0.05 considered significant

Category		No	Yes	*P* value
Year established				<.001
	Before 1981	8 (19.51)	131 (85.06)	
	Since or after 1981	33 (80.49)	23 (14.94)	
Has home rad onc program?				<.001
	No	40 (97.56)	41 (26.62)	
	Yes	1 (2.44)	113 (73.38)	
Has a general oncology interest group?				0.004
	No	30 (73.17)	71 (47.97)	
	Yes	11 (26.83)	77 (52.03)	
Has Oncology lecture during MS1-2?				0.004
	Yes	41 (100.00)	154 (100.00)	
Has a RO interest group?				0.050
	No	40 (97.56)	128 (86.49)	
	Yes	1 (2.44)	20 (13.51)	
Has a RO elective during MS3-4?				<.001
	No	26 (63.41)	48 (31.17)	
	Yes	15 (36.59)	106 (68.83)	
Has a RO oncology lecture during MS1-2?				0.207
	No	41 (100.00)	145 (94.77)	
	Yes	0 (0.00)	8 (5.23)	
Has career advisor for RO?				<.001
	No	38 (92.68)	29 (18.83)	
	Yes	3 (7.32)	125 (81.17)	
Has a home radiation oncology with radiation oncology residents?				<.001
	No	40 (97.56)	69 (44.81)	
	Yes	1 (2.44)	85 (55.19)	

## Discussion

Our study demonstrates the variability of RO education and experience in undergraduate medical training. Although all medical school curricula have oncology-based lectures, very few schools have a RO-specific lecture. Previous studies have demonstrated little to no involvement of radiation oncologists in the didactic portion of the undergraduate medical experience [4,7]. A recent survey demonstrates 60.8% of medical students had no exposure to RO [8]. This presents immediate area of improvement for our field. There was a weak association between pre-clinical RO education with home RO departments residents, which may demonstrate a special interest in education in these departments [3]. More studies are necessary to understand the influence of home RO programs on curriculum development during the first two years of medical school.

Furthermore, findings suggest significant differences between the allopathic and osteopathic medical schools were found. Students at osteopathic medical schools have less exposure to RO both in their pre-clinical and clinical years. Limited RO exposure and the increased competiveness of RO may explain these significant difference [9].

Mentorship remains a mainstay of RO, and the availability of RO career advising was found to have an association with the availability of RO electives and having students who match into a RO residency [10]. However, more recently established medical schools might have a disadvantage due to a lack of experience in RO and/or lack of an established RO department. The partnership between well-established medical schools and newer schools may benefit both entities for the promotion of RO. Additionally, purposeful growth of national mentorship programs in RO can help improve this gap.

To improve RO didactic education, oncology education as a whole should be improved [11]. Studies have established that oncology-related education remains under-emphasized compared to other topics, and medical students are not as comfortable with oncology when compared to other disciplines [5]. Medical schools may need to retool oncology education for the modern medical students due to the interaction of cancer with other fields. Furthermore, RO departments with both academic and private practice providers should reach out to their medical schools to help develop curriculum suitable for all medical students. Previous studies have demonstrated good outcomes with this option [6]. Due to the increase of new medical schools, national RO organizations such as ASTRO and ACRO can develop universal lectures that can be used by medical schools to improve curriculum, especially if they don’t have an established home RO department.

In conclusion, RO education during the didactic portion of the undergraduate medical experience remains extremely limited. This limitation is even more pronounced in medical schools without RO mentorship and in osteopathic medical schools. This lack of RO exposure perpetuates itself by bringing less students into the field. These issues require attention both on a national and medical-school-specific level.

## Data Availability

The authors declare that they had full access to all of the data in this study and the authors take complete responsibility for the integrity of the data and the accuracy of the data analysis.

## Previous presentation

Part of this work was presented at 2020 American Society for Radiation Oncology (ASTRO) annual meeting
